# Redox-sensitive small GTPase H-Ras in murine astrocytes, an *in vitro* study

**DOI:** 10.1080/13510002.2022.2094109

**Published:** 2022-07-13

**Authors:** Candida Zuchegna, Antonio Porcellini, Samantha Messina

**Affiliations:** a Dipartimento di Biologia, Complesso Universitario di Monte Sant'Angelo, Università degli Studi di Napoli “Federico II”, Napoli, Italia; b Dipartimento di Scienze, Università degli Studi Roma Tre, Roma, Italia

**Keywords:** Small GTPase Ras, murine astrocytes, redox signaling, GFAP

## Abstract

**Background:**

Although the protooncogenes small GTPases Ras are redox-sensitive proteins, how they are regulated by redox signaling in the central nervous system (CNS) is still poorly understood. Alteration in redox-sensitive targets by redox signaling may have myriad effects on Ras stability, activity and localization. Redox-mediated changes in astrocytic RAS may contribute to the control of redox homeostasis in the CNS that is connected to the pathogenesis of many diseases.

**Results and Methods:**

Here, we investigated the transient physiological induction, at both transcriptional and translational levels, of small GTPases Ras in response to redox stimulation. Cultured astrocytes were treated with hydrogen peroxide as in bolus addition and relative mRNA levels of murine *hras* and *kras* genes were detected by qRT-PCR. We found that *de novo* transcription of hras mRNA in reactive astrocytes is redox-sensitive and mimics the prototypical redox-sensitive gene iNOS. Protein abundance in combination with protein turnover measurements by cycloheximide-chase experiments revealed distinct translation efficiency, GTP-bound enrichment, and protein turnover rates between the two isoforms H-Ras and K-Ras.

**Conclusion:**

Reports from recent years support a significant role of H-Ras in driving redox processes. Beyond its canonical functions, Ras may impact on the core astrocytic cellular machinery that operates during redox stimulation.

## Introduction

RAS isoforms are not ubiquitously expressed, and their concentration depends on the organ and tissue type. RAS proteins share very similar molecular structures and they continuously cycle between GTP- and GDP-bound. Nevertheless, there is increasing evidence that they might exert different biological functions. Ras is a small G-protein highly expressed in the CNS, however there is limited information regarding its physiological roles. Astrocytes are particularly adapted to rapid detection of physiological changes in brain oxygenation [1]. Of note, the highest level of cellular RAS gene (in particular the *KRAS* gene) was detected in the brain mammalian tissues [2,3], albeit the protein levels in neural cells are unknown.

Various studies support regulation of Ras GTPases by reactive oxygen and nitrogen species [4–10]. Redox signaling is a form of signal transduction that progresses through the reversible oxidation of specific amino-acidic residues (mostly cysteines) in proteins mediated by hydrogen peroxide as a second messenger. Oxidation of these residues in many small GTPases affects the physiological processing of the proteins, the GTPase activity, the proper membrane localization and protein–protein interactions along signal transduction pathways [7].

Outside cancer, considerably less is known about the involvement of Ras in redox-based pathology, except for evidence on H-Ras in autoimmune diseases and diabetic retinopathy [11,12]. Hydrogen peroxide plays an important role in cellular physiology and is the key ROS (Reactive Oxygen Species) molecule in the development of multiple human diseases [13,14]. The involvement of redox signaling in reactive astrocytes is well established [15–17]. ‘Reactive astrocytes’ refer to astrocytes that respond to any pathological condition in the CNS when they become hypertrophic *in vivo* and overexpress the intermediate filament Glial Fibrillar Acidic Protein (GFAP). *In vitro,* we have previously shown that astrocytic Ras proteins mediate redox signaling in adaptive responses to redox stimuli [18]. Specifically, we previously demonstrated that serum withdrawal triggers H-Ras isoform in cultured astrocytes *via* ROS induction [18,19]. Moreover, interferon-γ treatment, the most potent inducer of ROS-RNS (Reactive Nitrogen Species) formation in target cells, induced transcriptional induction of *hras*, *nras*, and *kras* in cultured astrocytes [20] and adult human astrocytes stimulated with interferon-γ exert potent neurotoxicity *in vitro* [21]. Here, we report an *in vitro* characterization of mRNA abundance and protein turnover of cellular Ras proto-oncogenes by redox stimulation in mouse neocortical astrocytes.

## Results

### Redox-sensitive transcription of hras in primary astrocytes

**Figure 1.**
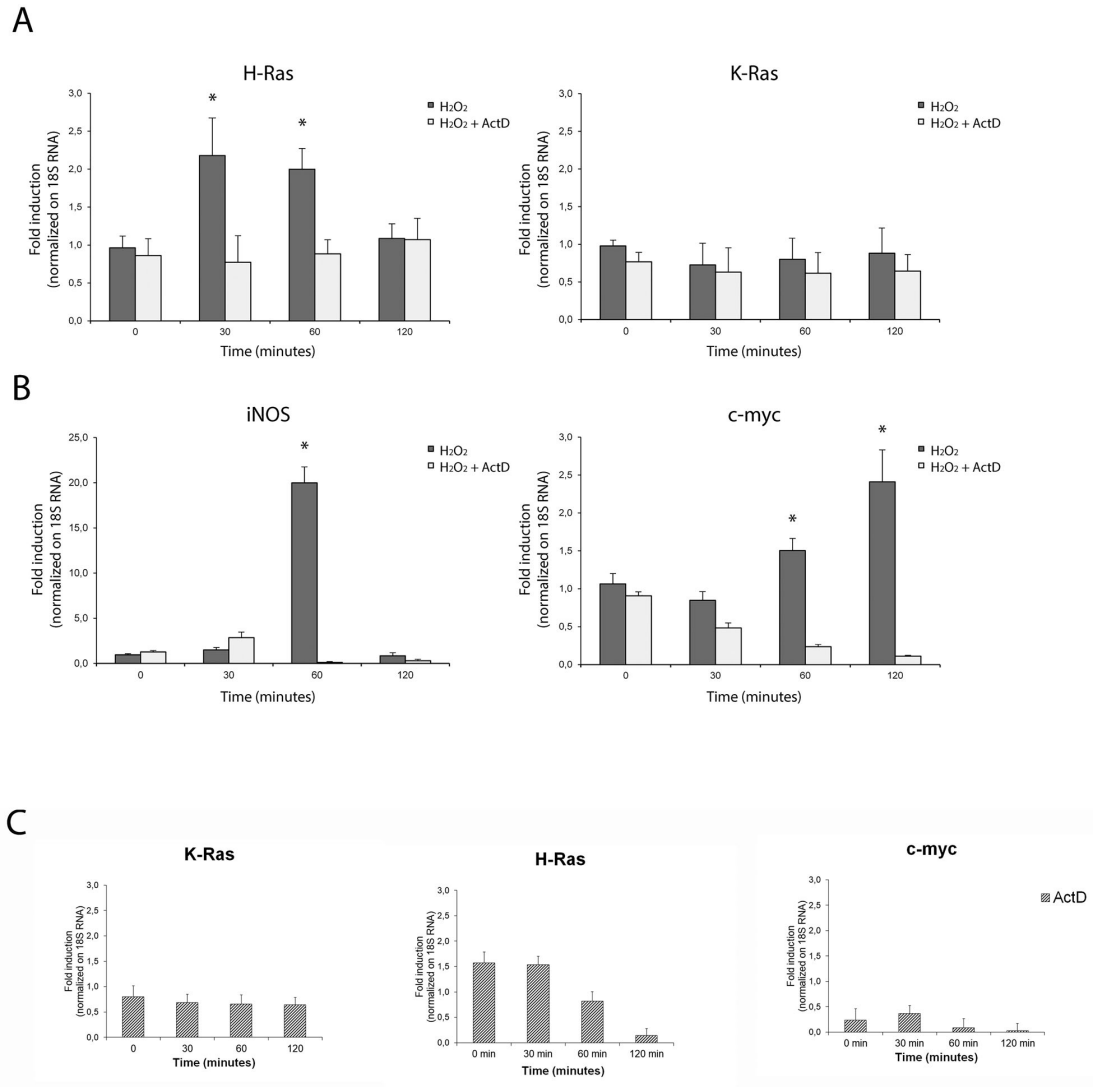
D*e novo* transcription of *hras* mRNA in reactive astrocytes is redox-sensitive. Relative mRNA expression levels by qPCR of *hras* and *kras* (A), i*NOS* and *c-myc* (B) genes in primary neocortical astrocytes challenged with 500 μM H_2_O_2_ for 30, 60 or 120 min in complete medium and in the presence or in the absence of Act D (10 μg/ml) pre-treatment. (C) qPCR analysis of astrocytic *hras, kras, iNOS, c-myc* treated with Act D alone as control (10 μg/ml; for 30, 60, 120 min). The mRNA levels are normalized to 18S RNA levels and the graph is plotted relative to untreated/growing value. Statistical analysis derived from four experiments in duplicate (*n* ≥ 8; mean ± SD); *P < 0.01 (matched pairs t-test) compared to untreated sample. See Supplementary Figure S1–S7 for statistical analysis raw data.

**Figure 2.**
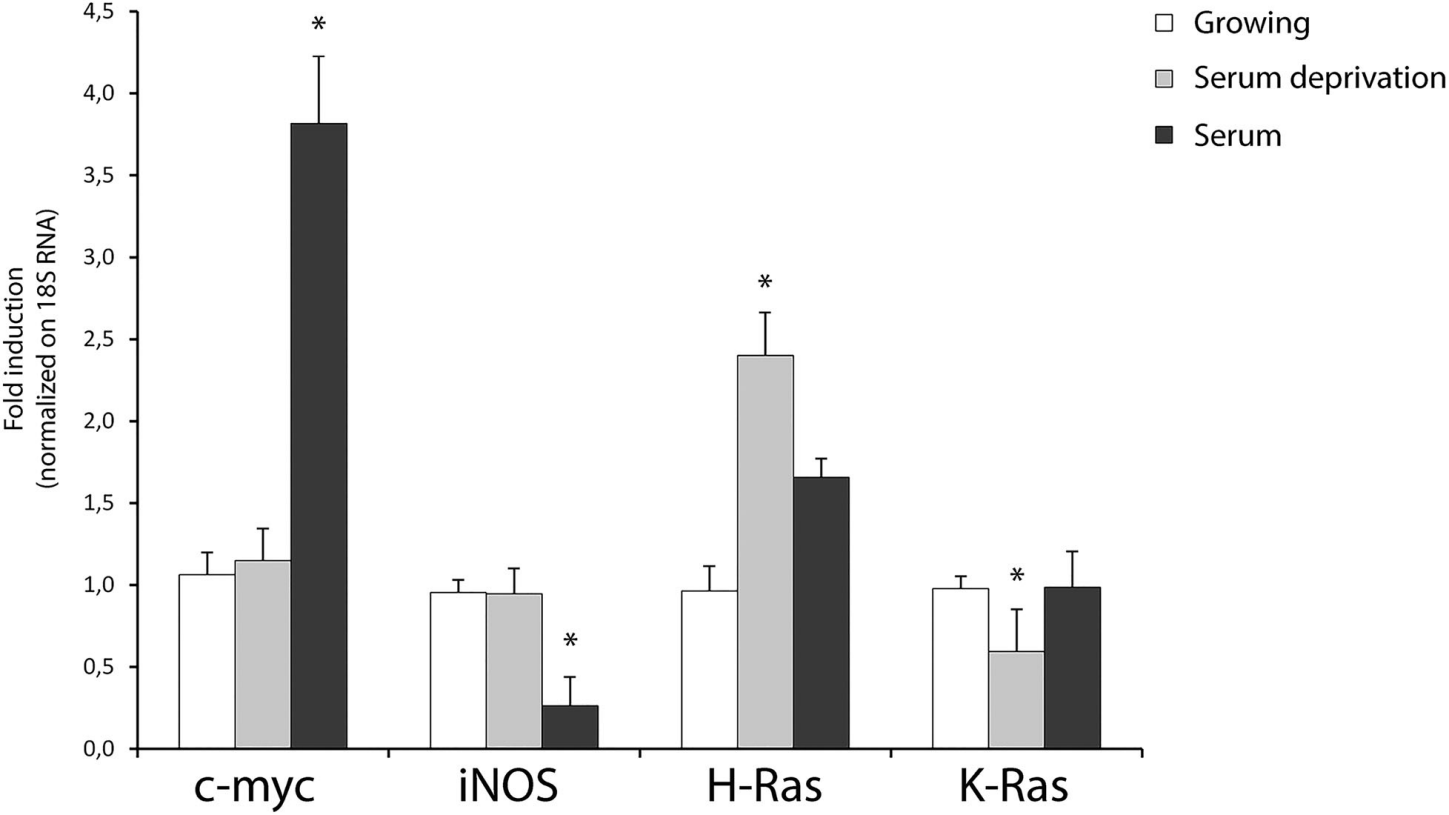
Quantitative transcriptional analysis by qPCR of proto-oncogenes *hras, kras, iNOS, c-myc* in primary neocortical astrocytes subjected to serum withdrawal (time = 3 h) and/or serum addition (time = 1 h). The mRNA levels are normalized to 18S RNA levels. Statistical analysis derived from four experiments in duplicate (*n* ≥ 8; mean ± SD); *P < 0.01 (matched pairs t-test) compared to untreated/growing value. See Supplementary Figure S1–S7 for statistical analysis raw data.

**Figure 3.**
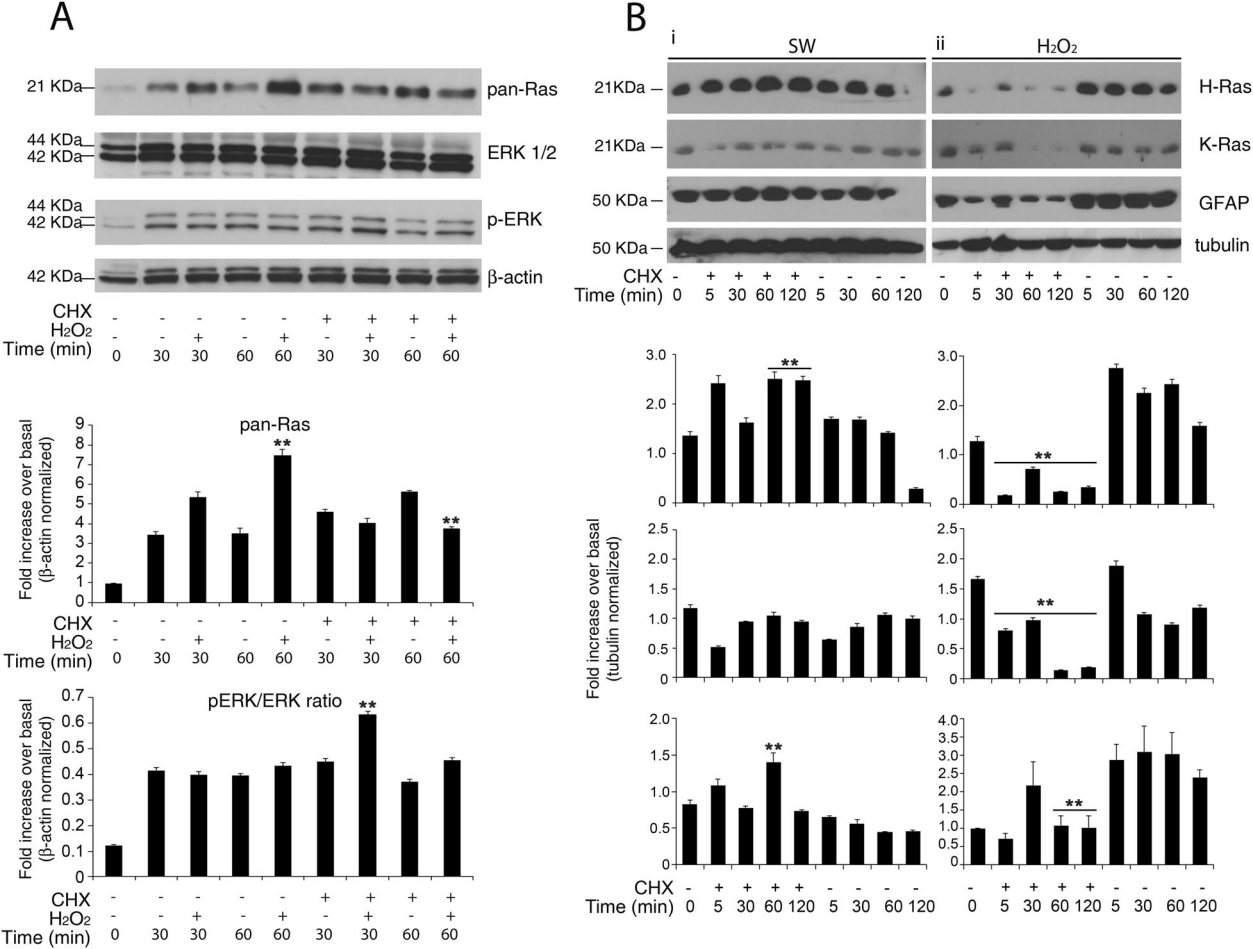
Redox-stimulation decreased protein turnover of H-Ras. (A) Representative immunoblot analysis of Ras in response to serum withdrawal or hydrogen peroxide administration. Acutely stimulated murine astrocytes were lysed with RIPA and immunoblot analysis with commercial antibodies against total Ras, ERK 1/2 and phosphorylated (p-) ERK ½ is shown. Growing primary astrocytes (15 DIV in 10% FCS serum) were subjected to trophic deprivation (0% FCS serum) or hydrogen peroxide administration (H_2_O_2_ 500 μM in complete serum) in presence or not of cycloheximide (CHX) pre-treatment for the indicated times. β-actin bands served as a loading control. Densitometric analysis is reported below (See Supplementary Figure S9 Raw data from densitometric analysis of western blot relative to Figure 3 panel A) (B) Astrocytes deprived of serum (i) or hydrogen peroxide treated (ii) were subjected to immunoblot analysis with the indicated antibodies anti-H-Ras Millipore #MAB3291; anti-K-Ras Sigma R3400; anti-GFAP #MAB3402 Clone GA5. Tubulin bands served as a loading control. Densitometric analyses of the Western blotting experiments results are reported as histogram above each panel and performed using Image Studio Light version 5. (See Supplementary Figure S10 and S14 Raw data from densitometric analysis of western blot relative to Figure 3) **P < 0.0001; *P < 0.01; °*P* < 0.05 as compared with the untreated astrocytes (Student’s t-test).

To examine whether redox stimulation is involved in the transcriptional induction of murine genes (*hras*, *kras*, *iNOS* and *c-myc*) in cultured astrocytes, we performed a quantitative reverse transcription PCR assay in the presence of actinomycin D (Act D) for measuring *de novo* transcription*.* mRNA was isolated from hydrogen peroxide-treated cultured astrocytes, and the abundance of *ras* transcripts was quantified by quantitative reverse transcription PCR (see Table 1 for murine primers in Methods). Hydrogen peroxide rapidly increased *h-ras* mRNA amount which was maximal at *t* = 30 min after stimulation and declined afterward (Figure 1 panel A). Transcriptional induction followed by translation accounted for the redox-induced H-Ras increase, as inhibition of gene transcription using 10 μg/ml Act D (Figure 1), and inhibition of protein synthesis using cycloheximide (CHX) (Figure 3) completely prevented the increase. Interestingly, we observed a concentration-dependent effect on the amount of *hras* mRNA induction, which was maximal with hydrogen peroxide low-doses (100 μM) compared to high-doses (500 μM) (data not shown). We therefore sought to exploit transcriptional regulation of inducible nitric oxide synthase iNOS in the same experimental conditions. iNOS mRNA raised the expression of twenty-fold over the basal and Act D pre-treatment completely abolished this increase at different concentration. Figure 1 panel B shows the kinetics of the inducible isoform Nitric Oxidase Synthase (*iNOS*) strongly upregulated by 500 μM of H2O2 at 1 h in neocortical astrocytes. Furthermore, the induction of the proto-oncogene *c-myc*, which is a short-lived mRNA between 0,5 and 1 h, was comparable in extent, but not in its kinetics, with *hras* mRNA. Of note, both Act D and cycloheximide treatments did not affect baseline H-Ras and K-Ras expression. Contrary to our previous results, no significant differences were detected in the *kras* mRNA transcript by redox stimulation (Figure 2).

**Table 1.** Complete list of murine DNA oligonucleotides used for qPCR.

	PRIMERS
iNOS Fw	5′-AGAGAGATCCGATTTAGAGTCTTGGT-3′
iNOS Rev	5′-TGACCCGTGAAGCCATGAC-3′
c-myc Fw	5′-TTTGTCTATTTGGGGACAGTG-3′
c-myc Rev	5′-CATCGTCGTGGCTGTCTG-3′
H-Ras Fw	5′-GTGACCTGGCTGGTCGCACTG-3′
H-Ras Rev	5′-CACTTGCAGCTCATGCAGCC-3′
18S Fw	5′-TCCCCATGAACGAGGAATTC-3′
18S Rev	5′-GGCCTCACTAAACCATCCAA-3′

As serum withdrawal (the main inducer of autophagy in astrocytes) results in the initial increase of intracellular ROS levels [22] and acute H-Ras induction in primary astrocytes [19], we measured the transcriptional response of these genes to two different stimuli (acute serum withdrawal and serum addition). Serum withdrawal was able to induce specifically *hras* isoform in cultured astrocytes, to downregulate *kras* mRNA and was ineffective with *iNOS* and *c-Myc.* Notably, serum addition sharply induced *c-Myc* mRNA (four-fold increase over the basal). These results indicate that *de novo* transcription contributes significantly to the H-Ras turnover by redox stimulation. We observed a linear correlation between mRNA and protein levels for H-Ras protein in our study. Conversely, redox induction of K-Ras protein is not transcriptional but probably mediated by differentially translated existing pools of mRNA [23].

### Redox-stimulation decreased protein turnover of H-Ras

Next, to discriminate the relative effects of translation on protein abundance of H-Ras and K-Ras, we used cycloheximide-chase assay (which is an inhibitor of protein biosynthesis due to its prevention in translational elongation) after single-time point western blotting (Figure 3). As previously reported, total Ras is up-regulated in response to both stimuli, hydrogen peroxide and serum withdrawal (Figure 3(A)). Hydrogen peroxide induces total Ras in a dose-dependent manner, exerting a maximal effect at 200–500 μM hydrogen peroxide ranging a plateau to 1–2 mM (Data not shown). Therefore, we search for a possible regulatory mechanism of ERK/MAPK cascade (which is an established target of Ras) by total Ras massive up-regulation. As predicted from the kinetics of total Ras expression, serum withdrawal and hydrogen peroxide activate the MAPK pathway cascade as assessed by phosphorylated ERK1/2 activation. The hydrogen peroxide-induced ERK activation shows a sustained induction over the entire time course that remains insensitive to the translational inhibitor cycloheximide. The maximal activation of the MAP kinases is detectable around 30 min and increased with cycloheximide (Figure 3(A) and Supplementary Figure S8).

To test isoform-specific differences in protein abundance during redox stress and serum withdrawal, we performed semiquantitative immunoblot analysis with specific antibodies against H-Ras and K-Ras. Acute inhibition of protein synthesis blunted both isoforms exclusively during redox stress (Figure 3(B) panel ii). Pre-treatment with the translation elongation inhibitor cycloheximide (CHX) (26) massively abolished the upregulation of H-Ras and K-Ras (Figure 3, panel ii). We also evaluated GFAP protein as the standard marker of astrocyte reactivity. Only a few *in vitro* studies have examined the half-life of wild type GFAP protein by cycloheximide chase-assay in cultured astrocytes. Here, we show that GFAP protein *in vitro* has monophasic decay with a short half-life of 60 min (Figure 3(B), ii and Supplementary Figure S8 and Supplementary Figure S15). Finally, redox stimulation requires active translation of H-Ras; otherwise serum withdrawal did not (Figure 3(B), i). One caveat for the cycloheximide block approach is that the protein half-life is measured when the overall protein synthesis is abrogated and, thus, may not reflect the actual turnover rate under normal growth conditions.

### Increase in GTP-bound H-Ras in reactive astrocytes by redox stimulation

**Figure 4.**
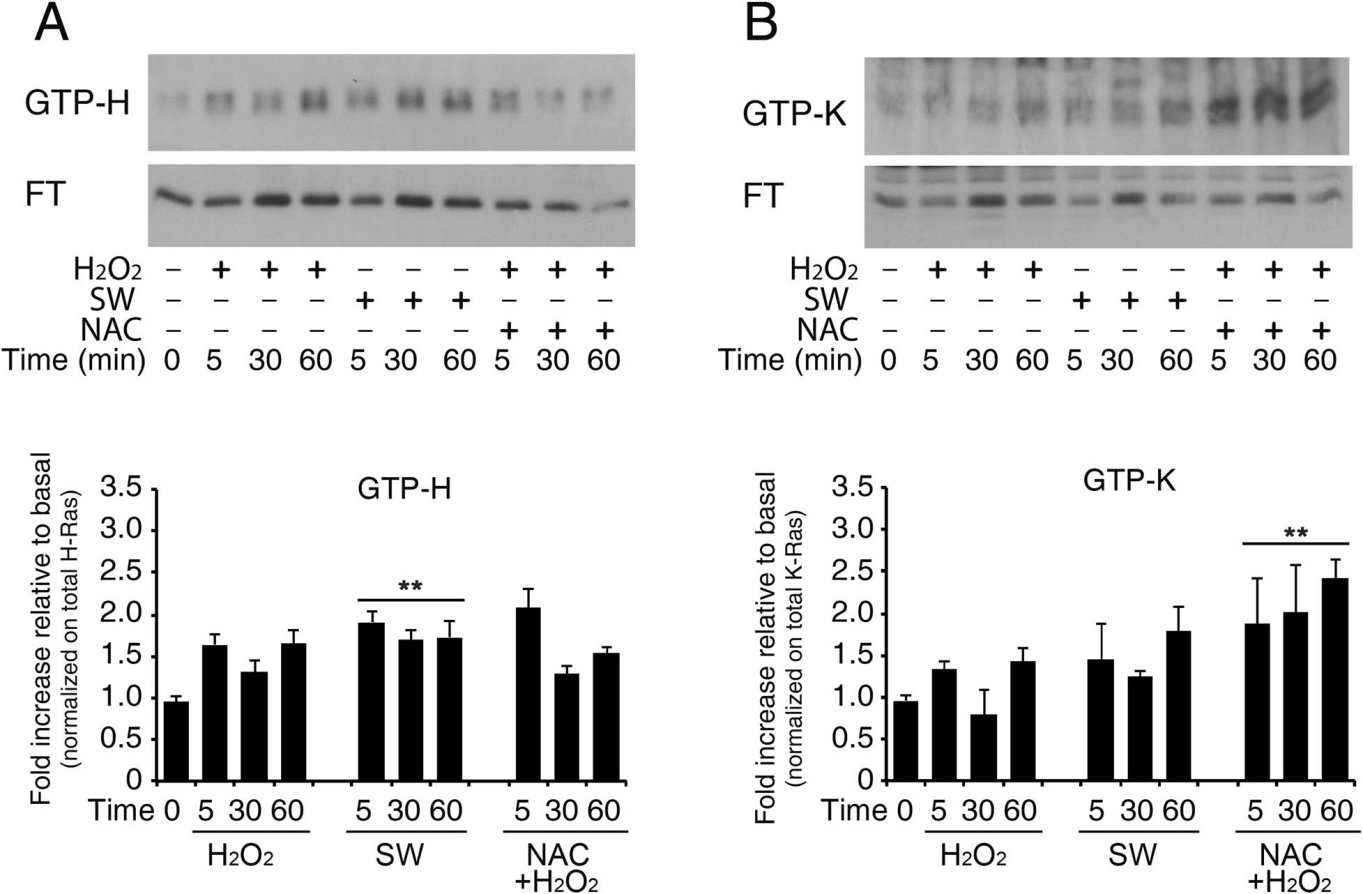
Increase in GTP-bound H-Ras in reactive astrocytes by redox stimulation. Acutely stimulated murine astrocytes were subjected to commercial c-Raf-1 RBD pull-down assay. Cells were grown and stimulated as indicated after or not pre-loading with the ROS scavenger N-Acetyl cysteine in complete serum (labels: SW, serum withdrawal; H_2_O_2_, hydrogen peroxide addition; NAC, N-Acetyl cysteine). H-Ras and K-Ras GTP-bound fractions were quantified by immunoblot after GST-RBD pull-down assay as described in Methods. Panel A shows immunoblot analysis of GTP-bound H-Ras and its flow-through (F.T.). The immunoblot is probed with the specific antibody anti-H-Ras (#MAB3291). Panel B shows immunoblot analysis of GTP-bound K-Ras and its flow-through (F.T.); the immunoblot is probed with the specific anti-K-Ras (Proteintech #12063-1-AP). Results represent the average of two independent experiments. Histogram representation of the quantitative densitometric analysis of GTP-bound fraction of each isoform and values are total Ras relative variation (see Supplementary Figure S14, Dataset Figure 4) **P < 0.0001; *P < 0.001; °*P* < 0.05 as compared with the untreated astrocytes (Student’s t-test).

Effects of exogenous redox agents on small GTPases perturb GTPase nucleotide-binding interactions that result in the enhancement of the Guanine Nucleotide Exchange of these enzymes [24]. To next evaluate the effect of redox stimulation on physiological GDP → GTP nucleotide exchange of Ras isoforms (H-Ras and K-Ras, but not N-Ras), we pulled-down active GTPase and detected them *via* Western blotting with specific antibodies. Active Ras Pull-Down assay revealed that the active H-Ras (GTP-bound H-Ras) level was upregulated in astrocytes in response to redox stimulation. This method traps all GTPases in the active form and results in GTP-bound enrichment. H-Ras–GTP and K-Ras-GTP were affinity sequestered by using glutathione transferase (GST)-Raf–Ras binding domain (RBD). The experiment was conducted also in the presence of N-Acetyl cysteine (NAC), a commonly used ROS scavenger. Representative immunoblot in Figure 4 shows GTPase enrichment preferentially for H-Ras isoform following hydrogen peroxide treatment (panel A) that was blunted by N-acetyl cysteine pre-treatment at 60 min. Furthermore, NAC pre-treatment causes a progressive reduction of total H-Ras between 5 and 60 min, as shown by the decrease in immunoreactivity in both flow-through (inactive form) and GTP-bound (active form). Conversely, K-Ras shows weak activation following treatment with hydrogen peroxide and an increase in its activated form (GTP-bound) in NAC-pre-treated samples. Under these conditions, total K-Ras is not reduced (Panel B) (see Figure S16 Uncropped versions of the western blot used in this manuscript). However, there are numerous pleiotropic effects associated with NAC that are often underappreciated.

## Discussion

Differences among Ras isoforms are increasingly appreciated in normal and neoplastic cells. Particularly, redox-sensitive small GTPases Ras show differences in isoform-distinct biological roles. In this paper we report an *in vitro* cellular H-Ras and K-Ras characterization at both transcriptional and translational level in mouse neocortical astrocytes. At transcriptional level, we observed a relatively fast *hras mRNA* increase by redox stimulation compared to *iNOS* gene that is sharply induced (twenty-fold increase over the basal). Consistent with our previous results, serum withdrawal induced specifically *hras* mRNA at higher extent than redox stimulation [19]. Other stimuli, such as serum addition or serum withdrawal, induced mRNA increase of *c-myc* gene in cultured astrocytes. Conversely, endogenous *kras* mRNA was poorly transcribed in reactive astrocytes. Accordingly, in humans *KRAS* is enriched for rare codons and codon usage regulates human *KRAS* expression at both transcriptional and translational level [25]. Moreover, the early response gene *c-myc* was sharply induced by both serum addition and hydrogen peroxide with a different kinetics.

Gene expression requires two steps – transcription and translation – which can be regulated independently to allow nuanced, localized, and rapid responses to cellular stimuli. Astrocytes, besides neurons, respond transcriptionally and translationally to bursts of brain activity [26]. Similarly, although the dynamic modulation of Ras GTPases is critically dependent on their post-translational modifications, burst of redox signaling in astrocytic cultures can induce a transcriptional mechanism. Here we show that both transcription and translation are active over the same period and contribute to the steady-state level of the H-Ras protein. Importantly, transcriptional-based regulation of RAS is less understood and to date there are no reports on such mechanisms in neuronal systems, to the best of our knowledge.

Redox signaling has been also shown to regulate the *iNOS* expression in different glial cells including astrocytes [27]. Furthermore, both oxidative stress and inflammation elicit the up-regulation of expression of iNOS in primary astrocytes [28]. The expression of iNOS in rat primary astrocytes and C6 glial cells has been described [29,30] and, specifically, hydrogen peroxide but not oxygen is actually required for astrocytic expression of iNOS *via* NF-κB [29]. Moreover, *ras* is involved in the transcription of iNOS in primary astrocytes [31]. Moreover, inducible nitric oxide synthase (iNOS) mRNA is induced in human astrocytes after oxygen-glucose deprivation (OGD). We can speculate that the transcriptional induction of astrocytic iNOS raises NO intracellular concentration that could contribute to Ras oxidation as largely reported in literature [10].

Moreover, H-Ras is activated by ROS that accelerates intrinsic nucleotide exchange, thereby promoting GTP loading [24]. Oxidative post-translational modification of Cys residues is an important mechanism that regulates Ras protein structure and ultimately function. It has been previously postulated that the specific amino-acid residue Cysteine 118 is conserved in N-, K-, and H-Ras due to its role in the small GTPases redox regulation [10,32,33], although cysteine residues modifications on endogenous Ras are uncertain. Anyway, ROS may facilitate the plasma membrane and/or endosome association of Ras, leading to its activation. As the GTP:GDP ratio in cells is approximately 10:1, oxidation-mediated GDP dissociation can promote GTP loading of Ras, analogous to the action of GEFs. Moreover, the oxidative modification may occur on reactive cysteine residues that are usually targeted for lipid-modification reactions. Thus, proper subcellular localization and physiological processing of the proteins could be antagonized by these redox-chemical modifications.

Reactive astrocytes are frequently identified by their strong expression of the intermediate filament protein GFAP, considered a universal marker for reactive astrocytes. Furthermore, we were surprised to notice that GFAP protein *in vitro* has monophasic decay with a short half-life of 60 min measured by CHX chase-assay, albeit this protein has a half- life of ≅3 days [35]. It should be noted that most knowledge about the biological, biochemical, and biophysical properties of GFAP is based on experimental data obtained with over-expressing GFAP astrocytes from transgenic mouse model of pathology. Importantly, in astrocytes, the GFAP protein needs vimentin to co-assemble into normal intermediate filaments [34] and in our experiment vimentin respond to the inhibitor of protein synthesis cycloheximide as such GFAP (data not shown).

## Limitations

The current study does have some limitations that should be mentioned. Our *in vitro* system (McCarthy-de Vellis astrocytes) [36] for the study of isolated astrocytes has several shortcomings. A major finding from expression profiling studies is that these astrocytes are highly dissimilar to mature astrocytes acutely purified from the healthy brain (astrocytes *in vivo*) [37,38]. Moreover, they are not representative of physiological astrocytes *in vivo* because they don’t maintain their quiescent state *in vitro*. These cultures grown in serum-containing media that is highly non-physiological, as most serum proteins are unable to cross the blood–brain barrier and likely profoundly alter astrocyte properties. Finally, human astrocytes exhibit greater susceptibility to oxidative stress than mouse astrocytes, due to differences in mitochondrial physiology and detoxification pathways.

## Conclusions

Despite significant progress in understanding some functional aspects of redox biology of Ras, the biochemical mechanisms underlying naïve proteins involvement in pathological and physiological contexts are not firmly established. This article focuses on the important and rather overlooked topic of redox signaling in astrocytes by examining the transcriptional and posttranscriptional regulation of Ras proteins in primary astrocyte cultures. The pivotal role of the astrocyte reactivity response in CNS injury and neurodegenerative disease has been established. In particular, Alexander disease is caused by astrocyte dysfunction due to a dominant mutation in GFAP and iNOS overactivation. Our findings raise the possibility that redox stress share pathophysiological mechanisms with Alexander disease and support Ras signaling as a potential therapeutic target. Nevertheless, a thorough and comprehensive understanding of the mechanism by which cause–effect relationships between GFAP regulation, iNOS activation and Ras regulation in astrocytes has to be explored.

## Methods

### Reagents and antibodies

Anti-Pan-Ras (Ab-3) Mouse mAb (RAS 10) #OP40 from Sigma-Aldrich Ltd; cycloheximide (CHX) and actinomycin D (Act D) were purchased from Calbiochem- Sigma-Aldrich Ltd (St Louis, MO, USA). The antibodies against ERK½, phospho-ERK½ were purchased from Santa Cruz Biotechnology. The anti-β-actin antibody and the Anti-c-K-Ras (clone 234-4.2) were from Sigma-Aldrich Ltd; Dnase I Improm II RT were purchased by Promega. Fetal Calf Serum (FCS) and Horse Serum, Trypsin-EDTA, and Penicillin/Streptomycin solutions were purchased from HyClone Europe Ltd; Modified Eagle’s Medium (MEM) and Dulbecco Modified Eagle’s Medium (DMEM), Trizol reagent and Lipofectin reagent from Life Technology. SYBR®Green and 2X Supermix cocktail were from Bio-Rad Laboratories Inc., Hercules, CA, USA. Anti-H-Ras Millipore #MAB3291; anti-K-Ras #R3400 Sigma-Aldrich Ltd (St Louis, MO, USA); anti-GFAP #MAB3402 Clone GA5 from Chemicon International. Goat anti-Mouse IgG1291 (H + L) Highly Cross-Adsorbed Secondary Antibody, HRP (A16078) e Goat anti-Rabbit IgG (H + L) Highly Cross-Adsorbed Secondary Antibody, HRP (A16119) Thermofisher Scientific. ECL detection kit from Amersham GE-Healthcare.

### Primary cultures of astrocytes

The cultures were performed as described previously in ref 32 with minor modifications. Cortices of postnatal day 0–3 (P0-P3) mouse pups of CD1 neonatal mice (Charles River Laboratories, Italy) were used for cortical astrocyte cultures. The protocol was approved by the local ethics committee. Briefly, after anesthesia with 5% isofluorane and then cervical dislocation, the brain was dissected, washed with PBS and the meninges were stripped off. The cortices were collected in growth medium (MEM supplemented with 10% fetal calf serum, 10% horse serum, 2 mM glutamine and 10.000 units/ml penicillin/streptomycin), washed in PBS, dissociated with 0.025% trypsin for 20 min at 37°C and then centrifuged (100 x g for 10 min at RT). The pellet was resuspended in growth medium containing 5 mM L-leucine methyl ester to limit microglia contamination and cells were plated (2 × 10^6^/10 ml) on 100-mm Falcon culture dishes and incubated at 37°C in atmosphere of 5% CO_2_. Confluent cultures contained >99% astrocytes and less than 0.1% microglia as shown by astrocyte staining with glial fibrillary acidic protein (GFAP). For half-life experiments, cultured astrocytes were pre-treated with cycloheximide (CHX) 20μg/ml in complete medium and then switching the cultures into low-serum (2%) medium containing H_2_O_2_. For qPCR experiments, cultured astrocytes were pre-treated with Act D at different concentration (5- 10 μM) and after washout switched the cultures into low-serum (2%) medium containing H_2_O_2_.

### qRT-PCR

cDNA was synthesized in a 20 μl reaction volume containing 1 μg of total RNA, 200 units of Superscript III Reverse Transcriptase (Invitrogen), and 2 μl random hexamer (20 ng/μl) (Invitrogen). mRNA was reverse-transcribed for 1 h at 50°C, and the reaction was heat inactivated for 15 min at 75°C. The products were stored at −20°C until use. The relative expression levels of selective genes were measured using SYBR Green (Invitrogen) reagent, and specific primers for analyzed genes were reported in Table 1. Commercially specific primers for murine *kras* gene were used according to the instructions (BioRad #100-25636). Primers were used at 250 nM final concentrations. Quantitative (q)RT–PCR were performed three times in six replicates on a 7500 RT–PCR System (Applied Biosystems) using the SYBR Green-detection system (FastStart Universal SYBR Green MasterRox/Roche Applied Science) as follows: *K-RAS* and 18S: 95°C 10 min; 40x (95°C 15 sec, 60°C 35 sec); *iNOS*: 95°C 10 min; 40x (95°C 15 sec, 60°C 1 min); *H-RAS*: 95°C 10 min; 5x (95°C 15 sec, 40°C 30 sec, 60°C 35 sec); 30x (95°C 15 sec, 60°C 1 min); 72°C 5 min; *c-MYC*: 95°C 10 min; 40x (95°C 40 sec, 55°C 30 sec, 72°C 30 sec); 72°C 10 min.

### Raf-1 Ras-binding domain-binding (RBD) assay

Cells were grown in 10-cm dishes, and were (or not) pre-treated with 5 mM N-Acetyl cysteine (NAC) for 2 h before H_2_O_2_ administration. H_2_O_2_ was added at the final concentration of 500 μM for 5, 30 or 60 min. After oxidative stimulus cells were harvested and Ras-GTP was identified by precipitation with GST-tagged Raf1-RBD with the Active Ras Pull-Down and Detection Kit (Thermo Scientific) following the manufacturer’s instructions. Briefly, cells were lysed in a buffer containing 25 mM Tris·HCl (pH 7.2), 150 mM NaCl, 5 mM MgCl_2_, 1% Nonidet P-40, 5% glycerol, and 1× protease inhibitor mixture (Roche Molecular Biochemicals). Lysates were clarified, the protein concentrations were normalized, and lysates (500 μg each) were incubated for 1 h at 4°C with 80 μg of GST-Raf1-RBD. Then GST beads were washed twice in the wash buffer. Samples were eluted and separated by SDS-PAGE electrophoresis in 15% gels and then transferred to nitrocellulose membranes (GE Healthcare, UK). Immunoblotting analysis was conducted with anti-c-K-Ras (clone 234-4.2) and anti-H-Ras Antibody (clone 7D7.2 MAB3291 Millipore). Moreover, as a positive loading control of the ability of GST-RDB resin to retain the GTP-bound form of Ras proteins, a fraction of the lysates was loaded with a stable non-hydrolysable GTP analog (GTP-γS, guanosine 5′-O-[gamma-thiol] triphosphate) following the manufacturer’s instructions.

### Western blot analysis

The assay was performed as described previously in ref 18 and 19 with minor modifications. Cultured astrocytes were washed with PBS and lysed for 15 min in ice-cold RIPA buffer (1% Triton X-100, 0.5% deoxycholate (DOC), 0.1% SDS, 50 mM Tris pH 7,6 150 mM NaCl, 1 mM PMSF, 1 mg/ml aprotinin, leupeptin and pepstatin). Cell lysates were clarified at 13,000 rpm for 30 min at 4°C and the cytosolic fraction was immediately frozen in liquid N_2_ for further analysis or subjected to immunoprecipitation procedures. Total protein concentration was determined using a Bradford colorimetric assay (Bio-Rad Laboratories Inc., Hercules, CA). Proteins were separated on SDS polyacrylamide gels (7–15%) and transferred onto nitrocellulose membranes. Blots were then blocked in Tris-buffered saline (50 mM Tris-HCl, 200 mM NaCl, pH 7.4) containing 5–10% non-fat dry milk (Bio-Rad Laboratories Inc., Hercules, CA) and incubated with primary antibodies (anti-pan-Ras antibody blocked in 5% non-fat dry milk for 2 h at room temperature, Ab-3 and mouse IgG2ak isotype anti-c-K-Ras, 1:500; anti- antibody, 1:400, all incubated overnight at 4°C; anti-β-actin blocked in 10% non-fat dry milk for 2 h at room temperature, 1:1,000, incubated 2 h at room temperature; anti-ERK1/2 and anti-phospho-ERK1/2, blocked in 10% non-fat dry milk for 2 h at room temperature (1:1,000), incubated 2 h at room temperature). Blots were washed three times with PBS and then incubated for 2 h with horseradish peroxidase-conjugated secondary antibodies (all used at 1:5,000). Immunostaining was revealed by the ECL detection system (Amersham). Densitometric analysis of Western data was carried out using a computer-based microdensitometer (NIH Image Software, Bethesda, MD).

### Ethics statement

CD1 mice (Charles River, Italy) were used for the preparation of pure astrocyte cortical cultures, as previously described [19]. All the experiments were performed in accordance with the named institutional body concerned with the ethics of experimentation (Italian ISS, Istituto Superiore di Sanità., Italy). All experiments were carried out in accordance with the European Directive of 22 September 2010 (EU/63/2010) and were authorized by the Italian Ministry of Health. All experimental protocols were approved by the Neuromed Institute Ethical Committee and subsequently approved by the Italian Ministry of Health.

### Statistical analysis

All data are presented as mean ± standard deviation. Data sets were analyzed statistically using the JMP Statistical Discovery™ software 6.03 by SAS (Statistical Analysis Software) and tested for normal distribution of variables using the Shapiro-Wilks test (‘normal distribution fit’ tool – JMP software). Statistical significance between groups was determined using Student’s t test, one-way analysis of variance (ANOVA), or multivariate analysis of variance (MANOVA) for normally distributed values, Wilcoxon/Mann–Whitney U test or Kruskal–Wallis test for not normally distributed data. Two-tailed significance tests were performed with *p* < 0.05 considered significant.

## Data Availability

The original contributions presented in the study are included in the Supplementary Materials. Further inquiries can be directed to the corresponding author.

## Abbreviations

Abbreviations: GFAP: Glial Fibrillar Acidic Protein; GDP: guanosine-5′-triphosphate; GTP: guanosine-5′-triphosphate; eNOS: endothelial nitric oxide synthase; iNOS: inducible nitric oxide synthase; NO: nitric oxide; RNS: reactive nitrogen species; ROS: reactive oxygen species; RT–PCR: reverse-transcription polymerase chain reaction; CHX: cycloheximide; ActD: actinomycin
